# Voltammetric Behavior, Flavanol and Anthocyanin Contents, and Antioxidant Capacity of Grape Skins and Seeds during Ripening (*Vitis vinifera var. Merlot*, *Tannat*, and *Syrah*)

**DOI:** 10.3390/antiox9090800

**Published:** 2020-08-27

**Authors:** Nawel Benbouguerra, Tristan Richard, Cédric Saucier, François Garcia

**Affiliations:** 1 SPO, Université de Montpellier, INRAE, Montpellier SupAgro, 34000 Montpellier, France; nawel.benbouguerra@etu.umontpellier.fr (N.B.); cedric.saucier@umontpellier.fr (C.S.); 2 MIB, Unité de Recherche Oenologie, EA4577, USC 1366 INRA, ISVV, Université de Bordeaux, 33882 Villenave d’Ornon, France; tristan.richard@u-bordeaux.fr

**Keywords:** skins, seeds, *Vitis vinifera*, antioxidant activity, cyclic voltammetry, phenolic compounds

## Abstract

Skin and seed grape extracts of three red varieties (Merlot, Tannat, and Syrah) at different stages of ripening were studied for their total phenolic content (TPC) by using the Folin-Ciocalteu assay and for their total antioxidant capacity (TAC) by using spectrophotometric and electrochemical assays. Flavanol and anthocyanin compositions were also investigated using Ultra Performance Liquid Chromatography coupled with Mass Spectrometry (UPLC-MS). Results showed that seeds had the highest phenolic content and the highest antioxidant potential compared to skins at all stages of ripening. The highest TPC and TAC values were measured in seeds at close to veraison and veraison ripening stages. In skins, the highest values were found at the green stage, it was in accordance with the flavanols content. The voltammetric measurements were carried out using disposable single walled carbon nanotubes modified screen-printed carbon electrodes (SWCNT-SPCE). Three peaks on voltammograms were obtained at different oxidation potentials. The first anodic peak that oxidized at a low potential describes the oxidation of ortho-dihydroxy phenols and gallate groups, the second peak corresponds to the malvidin anthocyanins oxidation and the second oxidation of flavonoids. The third voltammetric peak could be due to phenolic acids such as *p*-coumaric acid and ferulic acid or the second oxidation of malvidin anthocyanins. The high linear correlation was observed between antioxidant tests and flavanols in skins (0.86 ≤ *r* ≤ 0.94), while in seeds, ‘*r*’ was higher between electrochemical parameters and flavanols (0.64 ≤ *r* ≤ 0.8).

## 1. Introduction

*Vitis vinifera* is the most economically important species of grape vine in the world with 78 million tons of grapes production in 2018 (see http://www.oiv.int/en/oiv-life/oiv-2019-report-on-the-world-vitivinicultural-situation). Grapes consumed as fresh fruits, juices, and other processed products, contain many phenolic compounds which are mostly located in seeds and skins [1]. These compounds are synthesized in response to various biotic and abiotic stress such as fungal invasion, UV irradiations, ozone, and heavy metal ions [2]. Their content changes depending on the grape variety, soil, climatic conditions, and the ripening stages [3].

Polyphenols are commonly present in the plant kingdom and they bring more and more interest [4]. Phenolic compounds can be divided in two groups, flavonoids and non-flavonoids, according to their carbon skeleton [4]. The flavonoids (C6-C3-C6) are located in both skins and seeds and the anthocyanins and flavanols are the most abundant compounds [5]. The non-flavonoids such as stilbenes and phenolic acids are found in the skins [6]. The synergy between the various classes of polyphenols increases sample efficiency and activity [7]. Polyphenols protect plants against biotic and abiotic stresses and they are involved in organoleptic and qualitative properties of food and beverages derived from these plants [8]. Many studies have reported their biological activities. They have potent antioxidant capacity [7,9,10,11,12,13,14,15,16,17,18]. They may prevent diabetes [19,20], obesity [21,22,23], cardiovascular [24,25], and neurodegenerative diseases [25,26].

Radical scavenging capacity (DPPH and ABTS) and ferric reducing capacity, which are spectrophotometric assays, are usually used in order to determine the antioxidant capacity of foods and beverages [13]. In the last years, electrochemical techniques have been more widely used as alternative methods due to their sensitivity, rapidity, ease of use, and due to their minimal environmental effects [27]. Among these electrochemical techniques, cyclic voltammetry (CV) was the first and the most commonly used to characterize and determine the total polyphenols and the total antioxidant capacity [27]. The main CV (anodic curve) parameters are:

− The peak current which is proportional to the concentration of antioxidant.
− The peak potential which indicates the type of reductant (the more the oxidation potential is low, the more the reductant is strong and easy to oxidize).
− The charge (area under the curve) is in accordance with the antioxidant capacity of samples [28].

Electrode made of glassy carbon electrode is widely used but recently, carbon nanotubes electrode have become one of the most promising material [29]. This electrode is classified into two categories depending on the number of layers on multi-walled carbon nanotubes (MWCNTs) and single-walled nanotubes (SWCNTs) [29,30]. Actually, disposable screen-printed carbon electrodes modified with carbon nanotubes attract the attention of many researchers because of their numerous advantages including disposability [31], reproducibility, practicality, high sensitivity, the ability to be miniaturized to minimize the consumption of samples, and the low detection limits [32,33].

The aims of this work were:

- To determine the polyphenol content of skin and seed extracts (Merlot, Tannat, and Syrah) during ripening.
- To measure the antioxidant capacity (DPPH, ABTS, and FRAP) of these extracts.
- To determine the cyclic voltammetry behavior of these extracts by using disposable single walled carbon nanotubes electrodes for electrochemical tests.
- To determine the correlations of electrochemical parameters with the other antioxidant assays as well as with the phenolic contents.

## 2. Materials and Methods 

### 2.1. Chemicals and Reagents

Folin-Ciocalteu reagent, sodium carbonate, 2,2’-azino-bis(3-ethylbenzothiazoline-6-sulfonic acid) diammonium salt (ABTS), persulfate de potassium, 1,1-diphenyl-2-picrylhydrazyl free radical (DPPH), sodium acetate trihydrate, ferric chlorure, 2,4,6-tri(2-pyridyl)-s-triazine (TPTZ), iron(II) sulfate heptahydrate, phloroglucinol, ascorbic acid, sodium acetate, tartaric acid, sodium hydroxide, gallic acid, trolox, catechin, caffeic acid, *trans*- resveratrol, hydrochloric acid, and glacial acetic acid were purchased from Sigma-Aldrich (Saint-Quentin Fallavier, France). Oenin chloride was obtained from Extrasynthese (Genay, France). Acetonitrile, methanol, and water UPLC-MS were purchased from Biosolve Chimie (Dieuze, France) and trifluoroacetic acid from Carlo Erba Reagents (Peypin, France).

### 2.2. Samples 

Three *V. vinifera* varieties (Merlot, Tannat, and Syrah) were harvested on 2017 at different stages of ripening: Green stage (GS), close to veraison (CV), veraison (V), and maturity (M) (Supplementary Data, Table S1) from INRAe experimental vineyard (Montpellier, France) (coordinates: 43°37′02.7″ N 3°51′22.3″ E, average annual temperature: 15.85 °C, average annual precipitation: 629 mm (the weather was quite dry), and soil: Gravels and river sand). The whole grapes were stored at −80 °C in plastic bags until polyphenols extraction.

### 2.3. Samples Preparation

Seeds and skins of thirty Merlot, Tannat, and Syrah berries were manually removed from the pulp. The polyphenols were extracted with 100 mL of acetone/water (70/30 *v*/*v*) deoxygenated with nitrogen for 5 min. The solutions were filtered through a 0.45 µm filter paper after stirring during 18 h in the dark, and they evaporated in a rotavapor under low pressure at 37 °C. The resulting products were freeze-dried and stored at −20 °C until their use in antioxidant and other analytical assays [34]. Three biological replicates were done. After extraction, skin and seed extracts were weighted (dry weight: DW) and they were stored at −20 °C between 5 and 12 months before being used in the experiments.

### 2.4. Determination of Phenolic Composition 

#### 2.4.1. Flavanols

The assay on flavanols was performed as described by [35]. Briefly, a solution of 0.1 N HCl in MeOH, containing 50 g/L phloroglucinol and 10 g/L ascorbic acid was prepared. Seed and skin grape extracts were dissolved in methanol and reacted for 20 min at 50 °C in this solution, and then combined with five volumes of 40 mM aqueous sodium acetate to stop the reaction.

The UPLC system was a Waters Acquity (Saint-Quentin-en-Yvelines, France), with a photodiode array detector (PDA), LC pump, and an auto sampler. The column used was a reversed phase UPLC with an Acquity UPLC BEH C18 column (2.1 × 50 mm, 1.7 μm particle size) (Saint-Quentin-en Yvelines, France). The method used a binary gradient with mobile phases: Mobile phase A containing 1% *v*/*v* aqueous trifluoroacetic acid and mobile phase B containing acetonitrile. The 20 min elution method at flow 0.45 mL/min was 0 min 2% B, 8 min 6% B, 14 min 20% B, 14.1 min 99% B, 16 min 99% B, 16.1 min 2% B, and 20 min 2% B. The column temperature was 40 °C. Eluting peaks were monitored at 280 nm. The catechin calibration curve was used. Results were expressed as mg/g of DW.

#### 2.4.2. Anthocyanins

Skin grape extracts were solubilized in MeOH/water (80/20 *v*/*v*) at an appropriate concentration then injected directly after filtration as described previously with some modifications [36].

The conditions of the chromatographic apparatus are the same as those mentioned in experimental Section 2.4.1. The column temperature was set at 50 °C. The 40 min elution method at flow 0.25 mL/min was 0 min 1% B, 5 min 8.8% B, 30 min 20.6% B, 30.5 min 96% B, 34 min 96% B, 34.1 min 1% B, and 40 min 1% B. The detection was monitored at 520 nm. The malvidin-3-O-glucoside calibration curve was used. Results were expressed as mg malvidin-3-O-glucoside equivalent (M3GE)/g of DW.

### 2.5. Determination of Total Phenolic Content 

Skin and seed grape extracts (dry weight) were solubilized in methanol at a concentration of 5 g/L. The same solution was used to determine the total phenolic content (TPC) and total antioxidant capacity (TAC) assays. To measure the absorbance, an Agilent Cary 60 UV-Vis spectrophotometer (Santa Clara, CA, USA) connected to the Cary win UV software was used.

The Folin-Ciocalteu method was used to determine the total phenolic content (TPC) [3,13,37]. Twenty μL of the diluted extract (see Section 2.5) and 100 μL of Folin-Ciocalteu reagent were added to 1.58 mL of water. After 30 s, 300 μL of sodium carbonate solution (20%) were added; the reaction mixture was thoroughly shaken and left for 120 min in the dark at room temperature (20 °C). Then, the absorbance was measured at 700 nm against the blank (sample was replaced by the methanol). The gallic acid calibration curve was used to determine the concentration of phenolic compounds in samples. The results were expressed as mg gallic acid equivalent (GAE)/g DW.

### 2.6. Determination of Antioxidant Capacities

#### 2.6.1. Radical Scavenging Activity: DPPH^•^ Assay

DPPH antioxidant capacity was determined according to a published protocol [38]. Fifty µL of diluted extract (see Section 2.5) was added to 1.95 mL of a DPPH (6 × 10^−5^ M) methanolic solution. After 30 min of incubation in the dark at room temperature (20 °C), the absorbance was measured at 515 nm. The trolox calibration curve was used. The results were expressed as µmol TE/g DW.

#### 2.6.2. Radical Scavenging Activity: ABTS Assay

ABTS antioxidant capacity was determined according to [39]. To generate ABTS^•^ radical, 20 mL of ABTS solution (7 mM) was added to 20 mL of a potassium persulfate solution (2.45 mM). The mixture was incubated at room temperature in the dark all night. The stock solution was diluted with water/ethanol (50/50 *v*/*v*) to an absorbance of 0.7 ± 0.02 at 734 nm. One hundred µL of diluted extract (see Section 2.5) was mixed with 1 mL of ABTS^•^ solution. After 10 min, the absorbance was measured at 735 nm. The trolox calibration curve was used. Results were expressed as µmol TE/g DW.

#### 2.6.3. Ferric-Reducing Antioxidant Power: FRAP Assay

FRAP antioxidant capacity was determined according to reference [40]. Fifty μL of diluted extract (see Section 2.5) and 150 μL of distilled water were added to 1.5 mL freshly prepared FRAP reagent (mixture of 10 volumes of a 300 mmol/L acetate buffer pH 3.6 with 1 volume of 10 mmol/L TPTZ in 40 mmol/L hydrochloric acid and 1 volume of 20 mmol/L ferric chloride). The solution was incubated at 37 °C for 4 min. Absorbance was measured at 593 nm. The FeSO_4_·7H_2_O calibration curve was used. Results were expressed as mmol Fe^+2^E/g DW.

#### 2.6.4. Electrochemical Apparatus and Measurements

Electrochemical measurements were carried out with potentiostat/galvanostat, Autolab PGSTAT 302N controlled by the Nova 2.1.3 software (Metrohm, Switzerland) in the personal computer (Supplementary Data, Figure S1). Tartaric acid buffer (3.3 mM tartaric acid adjusted with 1 M NaOH to obtain a pH 3.6) was used to prepare standard phenolic compounds solutions as well as diluted extracts (see Section 2.5) at appropriate concentrations (100 mg/L for skins and 20 mg/L for seeds). The scan rate was 100 mV/s.

**Disk Electrode**

Voltammetric measurements were carried out in a standard three-electrode electrochemical cell using an Ag/AgCl (KCl, 3 M) reference electrode, platinum counter electrode, and a glassy carbon electrode (GCE) of 3 mm diameter (Metrohm, Switzerland) as working electrode. Before each test, the working electrode surface was carefully polished with 3 µm alumina powder, then washed with purified water and cleaned for 5 min in an ultrasonic bath. 

**Disposable Single-Walled Carbon Nanotubes Electrodes**

Single-walled carbon nanotubes electrodes (4 mm diameter, Dropsens, Spain) were also used in a three-electrode configuration comprising single-walled carbon nanotubes (SWCNTs-SPCE) with a silver reference electrode and carbon counter electrode. An aliquot of 200 µL of a solution of standard polyphenols or samples was cast onto the surface of the electrode, and the electrochemical measurements were performed immediately.

### 2.7. Statistical Analysis

The ANOVA and correlation tests were calculated by using the XLSTAT software (Addinsoft version 19.02, Paris, France). A Tukey test was carried out and where *p*-values < 0.05 was considered as significant. Pearson’s correlation coefficient was carried out for the determination of correlations between the different antioxidant assays (spectrophotometric and electrochemistry) and between the antioxidant assays and phenolic composition (anthocyanins and flavanols).

## 3. Results and Discussion

### 3.1. Flavanol and Anthocyanin Content of Skin and Seed Grape Extracts during Ripening

The results of the evolution of total flavanols and anthocyanins content in skin and seed grape extracts are presented in Table 1.

#### 3.1.1. Flavanols

**Skins**

For the three varieties, the highest flavanol content was determined at the green stage then it decreased significantly until maturity. It declined from 224 mg/g DW at the green stage to 19 mg/g DW at maturity in Tannat grape extracts. A similar evolution was shown in the literature [41,42]. On the opposite, an increase of flavanols content during ripening was also observed in other study [43].

**Seeds**

The highest content of flavanols was reached at close to veraison compared to the green stage and the maturity for all varieties. It increased from 424 mg/g DW at the green stage to 530 mg/g DW at close to veraison, then it declined significantly to 382 mg/g DW at maturity in seed Tannat grape extracts. This evolution was in accordance with a previous study [41]. The decline of flavanols content was explained by the oxidation of these compounds after veraison [44].

Flavanols are present in both skins and seeds at all stages of ripening with an abundance in seeds [27,45]. It has been shown that in Syrah skins at maturity the content was about 250 mg/g DW and about 455 mg/g DW in seeds [46]. There is an important variability in the literature concerning the phenolic composition content due to the extraction solutions, methods, and unit used to express results. 

#### 3.1.2. Anthocyanins

The anthocyanin synthesis started at close to veraison and they accumulated until maturity in Merlot and Syrah skins, in Tannat skins, the anthocyanin synthesis started at veraison. The content increased from 2 mg M3GE/g DW to 22 mg M3GE/g DW at maturity in skin Merlot extracts. A similar evolution was reported in the literature [41,42,43,47,48,49,50]. In the case of Syrah, the anthocyanins content decreased at maturity from 28 to 14 mg M3GE/g DW, this decline may be due to the degradation of anthocyanins by the peroxidases and glycosidases present in skins [47].

Anthocyanins, the pigmented compounds, are present only in skin red grapes. As flavanols, the anthocyanins content differs considerably in the literature. It increases from 1.80 to 3.81 mg/g DW in Tannat skins [51] and it is about 86.68 mg/g DW at maturity in another study [16]. As mentioned previously, the anthocyanins content is also greatly affected by weather, climatic conditions, soil conditions, cultivars, irrigation [49], temperature, and light [52].

### 3.2. Electrochemical Behavior of Polyphenol Standards and Skin and Seed Extracts for Various Cultivars at Different Stages of Ripening

#### 3.2.1. Electrochemical Behavior of Standard Polyphenols

**Figure 1:**
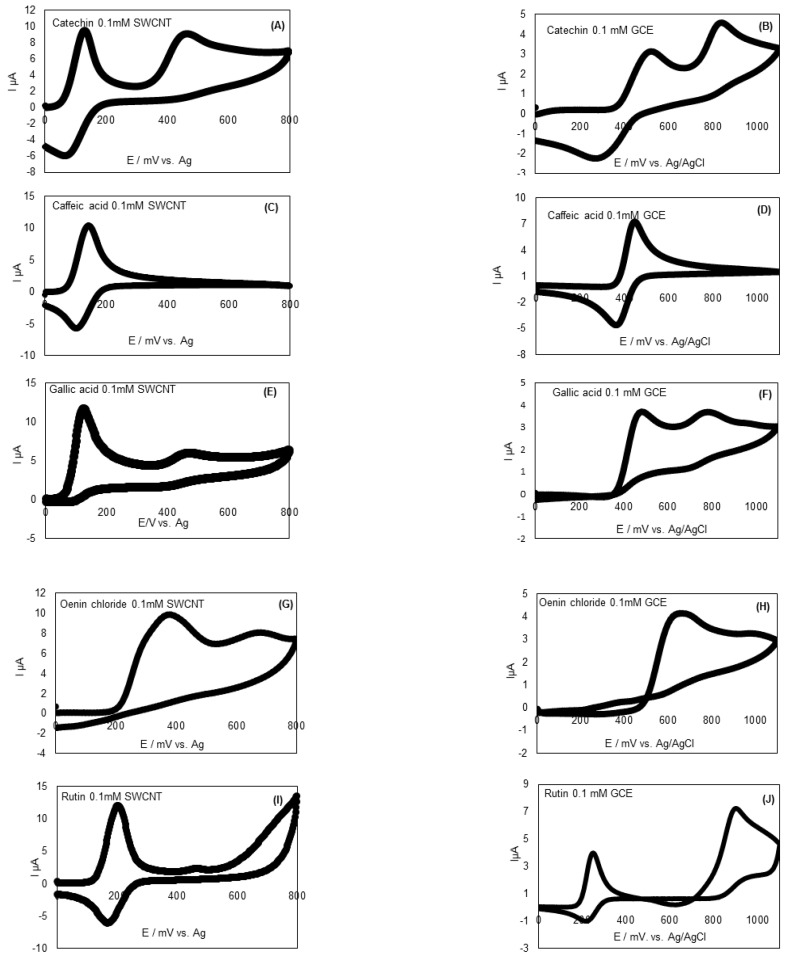
Cyclic voltammograms of catechin with SWCNT (**A**) and GCE (**B**), caffeic acid with SWCNT (**C**) and GCE (**D**), gallic acid with SWCNT (**E**) and GCE (**F**), oenin chloride with SWCNT(**G**) and GCE (**H**), rutin with SWCNT (**I**) and with GCE (**J**) at a concentration of 0.1 mM (blank subtracted). GCE: Glassy Carbon Electrode; SWCNT-SPCE: Single Walled Carbon Nanotubes modified Screen Printed Carbon Electrodes.

Cyclic voltammograms of polyphenol standards in tartaric acid buffer (pH 3.6) at glassy carbon electrode (GCE) in a potential range from 0 to 1100 mV (vs. Ag/AgCl-KCl 3M) and at single-walled nanotubes (SWCNT) in a potential range from 0 to 800 mV (vs. Ag) are illustrated in Figure 1 and peak potentials are given in Table 2. For caffeic acid, only one anodic peak was present. This peak corresponds to the oxidation of the *ortho*-diphenols to form the corresponding o-quinone. The potential values for the concentration 0.1 mM are 445 mV (vs. Ag/AgCl-KCl 3M) for GCE and 139 mV (vs. Ag) for SWCNT. Two peaks were observed for catechin and gallic acid at 0.1 mM. With GCE (vs. Ag/AgCl-KCl 3M), the voltage values were 483/826 mV for gallic acid and 472/766 mV for catechin. With SWCNT (vs. Ag), the voltage values were 132/468 mV and 122/465 mV for catechin and gallic acid, respectively. For both catechin and gallic acid, the first anodic peak correspond to the oxidation of the hydroxyl groups on the B-ring to quinone [53]. This oxidation was reversible generating cathodic peak in the negative scan for caffeic acid and catechin. The second peak corresponds to the oxidation of the hydroxyl group on the C-ring of catechin and can also correspond to the oxidation of the third phenol group adjacent to the ortho-diphenol group in gallic acid which is in agreement with previous results [54]. Other phenolic standards characterized corresponding to the anthocyanins and the flavonols are present mostly in skin grapes. Oenin chloride and rutin at 0.1 mM presented two anodic peaks at 377/669 mV and at 201/460 mV with SWCNT (vs. Ag), respectively, and at 652/987 mV and at 260/898 mV with GCE (vs. Ag/AgCl-KCl 3M), respectively (Figure 1).

The classification obtained considering only the first peak potential for the studied standards at the same concentration (0.1 mM) by increasing potential was: Gallic acid 122 (mV) < catechin (132 mV) < caffeic acid (139 mV) < rutin (201 mV) < oenin chloride (377 mV) was found [55] since catechin, caffeic, and gallic acid oxidized at lower potential.

#### 3.2.2. Electrochemical Characterization of Skins and Seeds 

**Figure 2:**
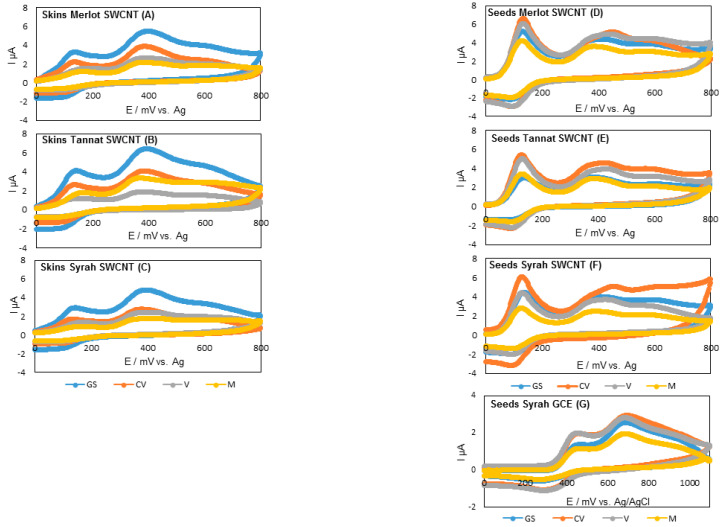
Cyclic voltammograms of skin (**A**–**C**) and seed grape extracts (**D**–**F**) with SWCNT and those of Syrah seed grape extract (**G**) with GCE at different stages of ripening (blank subtracted). GS: Green Stage; CV: Close to Veraison; V: Veraison; M: Maturity.

Voltammetric measurements were performed on the extracts of each variety. For all varieties, cyclic voltammograms had three anodic peaks at different potentials depending on grape part (skins or seeds) (Figure 2) and the ripening stage. Syrah grape seed extracts were studied with both types of electrodes, as with SWCNT, three anodic peaks were also obtained with GCE (Figure 2).

For skin Merlot grape extracts, the first anodic peak was measured at 137, 134, 159, and 157 mV at the green stage, close to veraison, veraison, and maturity, respectively. This peak corresponds to the more oxidizable compounds that oxidized at a low potential as catechin-type flavonoids, including larger oligomeric and polymeric molecules, gallic acid, caffeic acid, and flavonols. The second anodic peak appeared at 391, 383, 363, and 370 mV at the green stage, close to veraison, veraison, and maturity, respectively. This peak may result from the oxidation of malvidin anthocyanins and stilbene derivatives overlapped with the second oxidation of the catechin flavonoids [56]. The third peak close to 600 mV corresponds to the phenolic acids such as vanillic and *para*-coumaric acid or the second oxidation of malvidin anthocyanins [54]. The same behavior was observed for the two other varieties.

In grape seed extracts, the first anodic peak was obtained at the same potential in all stages of ripening, it was around 130 mV. For Syrah grape extracts for example, the first peak appeared at 136 mV, 127, 129, and 126 mV at the green stage, close to veraison, veraison, and maturity, respectively. The second peak followed the same trend of the first potential with the following potential values for Syrah at 396, 438, 409, and 391 mV for the different stages of ripening. This peak could be attributed to the oxidation of the hydroxyl group on the C-ring of catechin derivatives. The third anodic peak corresponds to the higher oxidation potential compound which produces a peak at around 600 mV [57]. 

### 3.3. Total Phenolic Content and Total Antioxidant Capacity by Spectrophotometric and Electrochemical Assays

#### 3.3.1. Total Phenolic Content and Total Antioxidant Capacity by Spectrophotometric Assays

The total phenolic content and the antioxidant capacity of skin and seed grape extracts during ripening were determined using different spectrophotometric methods: Folin-Ciocalteu, DPPH, ABTS as well as FRAP assays, respectively. The results were summarized in Table 3.

**Skins**

The highest total phenolic content was detected at the green stage of ripening then it decreased significantly at maturity in the three varieties. For example, in Syrah grape extracts, the total phenolic content (TPC) was 212 mg GAE/g DW at the green stage then declined to 63 mg GAE/g DW at maturity. 

The antioxidant capacities were measured using a single electron transfer (DPPH, ABTS, and FRAP). The highest total antioxidant capacity (TAC) was found in the green stage compared with maturity. The same evolution was obtained with the three assays on the three varieties. For example, in the skin Syrah grape extract, DPPH values decreased significantly from 853 at the green stage to 557 µmol TE/g DW at maturity, ABTS values from 843 to 357 µmol TE/g DW, and FRAP values from 2159 to 780 mmol Fe^+2^E/g DW.

**Seeds**

The TPC in seed grape extracts increased before veraison then decreased after veraison with the highest content at close to veraison for both seed Tannat and Syrah grape extracts, whereas for seed Merlot grape extract, the content decreased significantly from 867 at the green stage to 571 mg GAE/g DW at maturity.

The antioxidant capacity of seed grape extracts followed the same trend with the three antioxidant assays. The antioxidant capacity at close to veraison was higher than that found at the green stage and maturity. Among the samples tested, for seed Syrah grape extracts, the DPPH values increased from 2677 to 2915 µmol TE/g DW then decreased to 1991 µmol TE/g DW. ABTS values raised from 1171 to 1325 then declined to 590 µmol TE/g DW. FRAP values increased from 3979 to 5386 mmol Fe^+2^E/g DW then decreased to 3460 mmol Fe^+2^E/g DW.

Several methods were used to determine total phenolic content and antioxidant capacity of samples to take into account not only the composition of the extracts but also the mode of action and the specificity of the antioxidant [58,59]. Due to its ease of use, the Folin-Ciocalteu assay is the common used method to determine the TPC. The principle is the transfer of electrons from phenolic compounds to phosphomolybdic/phosphotungstic complexes [60]. The weakness of this method is the overestimation of the phenolic content due to the lack of specificity [55,60,61] which can react with other compounds particularly aromatic amines, ascorbic acids, and sugars [61]. In addition, the phenolic compounds react with the Folin-Ciocalteu reagent only under the basic conditions [61]. The three colorimetric methods used to determine the antioxidant capacity DPPH, ABTS, and FRAP are considered as assays based on the electron transfer [58,61]. DPPH assay is an easy method widely used to determine the antioxidant capacity of natural extracts. The drawback of this method is the variation of reaction time with different phenolic compounds. Caffeic acid, for example, reacts quickly with DPPH whereas the catechin reacts slowly. The results obtained with this method differ depending on the time of readings (from 16 min to some hours) [55]. The FRAP assay is a simple, fast, and robust method used in the determination of the concentration of the most easily oxidized compounds [61]. It is based on the ability to reduce Fe^3+^ to Fe^2+^ quantified at 593 nm. Fe(III)/TPTZ reagent is more stable than DPPH^•^ and gives results in shorter times [55]. The ABTS assay is based on the reduction by an antioxidant of the generated blue/green ABTS^+^ [62]. DPPH and ABTS assays are the easiest to implement and yield the most reproducible results [58]. FRAP and DPPH methods are still used as they are the easy and accurate methods to measure the antioxidant activity [60]. 

The results of this work confirm that the total phenolic content in skins were lower than in seeds [13]. In skins, the highest antioxidant capacity was found at the green stage but a previous study [48] found the highest TAC at maturity. This difference may depend on the extraction method used but also on the protocol of the test. The total polyphenolic content increased when the berry weight decreased in accordance with previous studies [51]. 

#### 3.3.2. Antioxidant Capacity by Electrochemical Method of Skin and Seed Grape Extracts

Different parameters shown in Table 4 allowed the estimation of the antioxidant capacity of extracts by cyclic voltammetry. The total charge Q_800mV_ corresponds to all oxidizable phenolic compounds that will contribute to the total antioxidant capacity of the extract. Q_240mv_ represents the electrochemically of the easily oxidizable polyphenols that have consequently the highest antioxidant capacity. Q_520mv_ estimates the most antioxidant compounds which oxidize until 520 mV (until the second peak of the voltammogram). Q_520mv_-Q_240mv_ corresponds to the compounds that have the lesser antioxidant capacity that oxidize until 520 mV. Finally, Q_240mv_/Q_800mv_ ratio indicates the contribution of the most antioxidant compounds to the total antioxidant capacity of extract. 

**Skins**

Q_800mV_, Q_240mV_, and Q_520mV_ values presented the same evolution for all grape skin extracts. They declined from the green stage to maturity. For example, in Merlot, Q_800mV_ values decreased from 262 to 118 µC/g DW, Q_240mV_ from 44 to 22 µC/g DW, and the antioxidant capacity until 520 mV diminished from 153 to 75 µC/g DW. The contribution of the most antioxidant compounds to the total antioxidant capacity was also determined. It followed the same evolution of the other parameters except for Merlot grape extracts where the percentage increased from 17 to 22% then decreased to 19%.

**Seeds**

Electrochemical parameters of seed grape extracts have the same evolution in the three varieties. They raised from the green stage to close to veraison and veraison then declined until maturity. In Merlot, Q_800mV_ values increased from 1232 to 1471 µQ/g DW then decreased to 1036 µC/g DW at maturity. The antioxidant capacity at 240 mV was about 358 µC/g DW at the green stage, 379 µC/g DW of extract at veraison, and 252 µC/g DW at maturity. Antioxidant capacity of seed extracts until 520 mV has the same trend than the other parameters, it stated from 905 µC/g DW at the green stage then increased to 944 µC/g DW at veraison, and declined to 639 µC/g DW at maturity. The most antioxidant compounds almost contribute with the same percent at all stages of ripening except for Merlot where the percent of Q_240mV_/Q_800mV_ decreased from 30% to 24% at maturity.

Electrochemical parameters in both skins and seeds have the same trend than TPC, TAC values, and flavanol content. The higher TPC and TAC were found in seed grape extracts compared with skin grape extracts, in agreement with the literature [3,45,63]. The percent of Q_240mV_/Q_800mV_ in seed grape extracts was more important than in skin grape extracts. It follows the same evolution of the other parameters in skins except for Merlot, in seeds there is no among differences between stages. At the charge Q_240mV_ corresponding to the oxidation of flavanols, this result suggests the abundance of these compounds in seeds compared with skins.

### 3.4. Correlation between TPC, Antioxidant Capacity, and Phenolic Composition 

Table 5 shows the Pearson correlation coefficients between TPC, electrochemical parameters, antioxidant assays, and phenolic composition for which: *r* < 0.39 weak correlation, 0.4 < *r* < 0.69 moderate correlation 0.7 < *r* < 0.89 strong correlation, and 0.9 < *r* < 1 very strong correlation [64].

In skins, a strong correlation was found between TPC and electrochemical parameters (*r* = 0.88 vs. Q_240mV_, *r* = 0.90 vs. Q_520mV_, and *r* = 0.84 vs. Q_800mV_). It was shown in the literature that TPC is significatively correlated with electrochemical responses [65], especially with cumulative response up to relatively high potentials [32]. In this study, TPC was better correlated with Q_240mV_ than Q_800mV_. Colorimetric antioxidant assays (DPPH, ABTS, and FRAP) were strongly correlated with all electrochemical parameters. The best correlation was found with Q_240mV_ (*r* = 0.81 vs. DPPH, *r* = 0.89 vs. ABTS, and *r* = 0.86 vs. FRAP) than with Q_800mV_ (*r* = 0.69 vs. DPPH, *r* = 0.69 vs. ABTS, and *r* = 0.75 vs. FRAP). The methods used are well correlated because they are all based on electron transfer from antioxidant to oxidized compounds [32]. Flavanols content were well correlated with colorimetric assays (*r* = 0.93 vs. Folin-Ciocalteu, *r* = 0.86 vs. DPPH, *r* = 0.86 vs. ABTS, *r* = 0.94 vs. FRAP) as well as electrochemical parameters (*r* = 0.87 vs. Q_240mV_, *r* = 0.72 vs. Q_800mV_). The strong correlation between flavanols and Q_240mV_ compared with Q_800mV_ indicates that these compounds are the easiest antioxidant compounds that oxidized at a low potential (240 mV). A negative correlation between anthocyanins and the antioxidant tests have been shown, this result is an agreement with a previous study [59].

In seed grape extracts, the best correlation was found between flavanols and electrochemical parameters (*r* = 0.80 vs. Q_240mV_, *r* = 0.80 vs. Q_520mV_, and *r* = 0.64 vs. Q_800mV_) than with spectrophotometric methods (*r* = 0.67 vs. Folin, *r* = 0.66 vs. DPPH, *r* = 0.71 vs. ABTS, and *r* = 0.58 vs. FRAP). A strong correlation was observed between Folin-Ciocalteu, DPPH, and ABTS (*r* = 0.78 vs. DPPH and *r* = 0.77 vs. ABTS) whereas a moderate correlation was found between Folin-Ciocalteu and FRAP (*r* = 0.67). Contrary to skin grape extracts, in seed grape extracts, FRAP have the lowest correlation with all assays compared with the other colorimetric methods. This result illustrates the specificity of each assay and the variability of phenolic composition between skin and seed grape extracts.

The antioxidant capacity was mainly related to the TPC of extracts in accordance with previous results [9,12,13,14,58,62,66,67] and especially to the flavanols content [14]. The antioxidant capacity of polyphenols is mainly linked to their structures, compounds that have more than one aromatic ring, more than one hydroxyl groups in different positions are able to have a highest antioxidant capacity. This may explain the variability of Pearson correlation between the different methods and between skins and seeds.

**Principal Components Analysis (PCA)**

**Figure 3:**
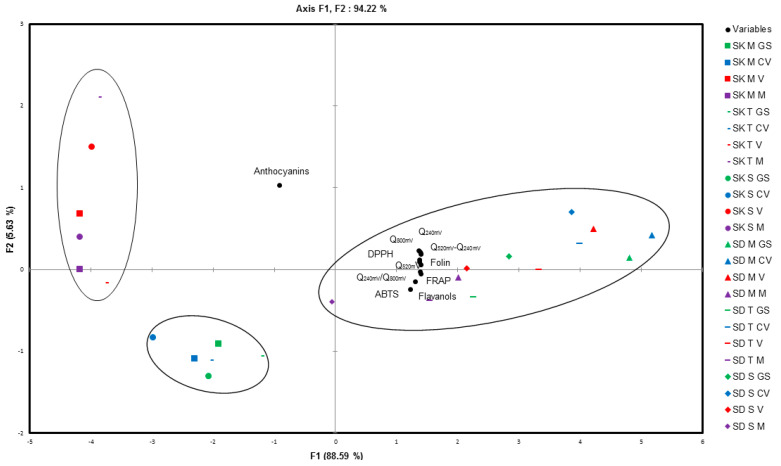
Biplot of the first PCs obtained from PCA for seeds (SD) and skins (SK) of three grape varieties Merlot (M), Tannat (T), and Syrah (S) at different stages of ripening (green stage (GS), close to veraison (CV), veraison (V), and maturity (M)).

Figure 3 shows the Biplot graphic that represents the association of the phenolic composition with the antioxidant assays on skin and seed grapes extracts during ripening. The first two principal components explained 94.2% of the total variability. The first axis accounted for 88.6% and the second axis only for 5.6%. From the Biplot, skin grape extracts are separated in the left side from seed grape extracts in the right side.

For skin grape extracts, the stages of maturity were well separated depending mainly on the content of anthocyanins, flavanols, as well as antioxidant capacity, down the stages before veraison (have the highest flavanols content and antioxidant capacity) and up the stages from veraison to maturity (beginning of synthesis and accumulation of anthocyanins, low antioxidant capacity, and flavanols content). For seed grape extracts, it is more difficult to separate the different stages of maturity because the variables are very close.

Flavanols were compounds with the highest positive contribution to the antioxidant capacity, while the anthocyanins were the highest negative contribution in the three varieties studied. As it can be seen in Figure 3, the content of flavanols and the antioxidant capacity were higher in seed than in skin grape extracts. 

## 4. Conclusions

Total phenolic content, antioxidant capacity, flavanol, and anthocyanin contents of grape skin and seed extracts of three red grape varieties were studied at different stages of ripening. At all stages of ripening, the total phenolic content was higher in seed than in skin grape extracts. The green stage had the highest total phenolic content in grape skin extracts, whereas in grape seed extracts, they were the close to veraison and the veraison that had the highest content.

To measure the antioxidant capacity of extracts, different colorimetric methods were used (DPPH, ABTS, and FRAP) in addition to cyclic voltammetry. In skin grape extracts, the total antioxidant capacity was higher at the green stage than at maturity, in seed grape extracts, they were the close to veraison and the veraison that had the highest content with all assays. Generally, stages that had the highest phenolic content presented also the highest antioxidant capacity.

The correlation between electrochemical results using disposable electrodes and the colorimetric assays indicates that electrochemical assays can be considered as an alternative to these routine tests in the determination and the characterization of the antioxidant capacity in a short time.

## Figures and Tables

**Table 1:** Phenolic composition of skin and seed grape extracts of the studied varieties at different stages of ripening.

	Skins		Seed
	Flavanols (mg/g DW)	Anthocyanins (mg M3GE/g DW)	Flavanols (mg/g DW)
Merlot			
Green stage	199 ± 19 ^a^	ND	545 ± 9 ^a^
Close to veraison	124 ± 16 ^b^	2 ± 1 ^c^	598 ± 30 ^a^
Veraison	45 ± 9 ^c^	17 ± 1 ^b^	437 ± 17 ^b^
Maturity	42 ± 2 ^c^	22 ± 1 ^a^	329 ± 24 ^c^
Tannat			
Green stage	224 ± 40 ^a^	ND	424 ± 27 ^bc^
Close to veraison	166 ± 74 ^a^	ND	530 ± 16 ^a^
Veraison	31 ± 5 ^b^	10 ± 3 ^b^	469 ± 21 ^b^
Maturity	19 ± 2 ^b^	36 ± 2 ^a^	382 ± 23 ^c^
Syrah			
Green stage	198 ± 34 ^a^	ND	496 ± 19 ^ab^
Close to veraison	100 ± 26 ^b^	3 ± 1 ^c^	532 ± 21 ^a^
Veraison	40 ± 7 ^c^	28 ± 1 ^a^	439 ± 32 ^b^
Maturity	18 ± 1 ^c^	14 ± 1.0 ^b^	201 ± 41 ^c^

Values represent means of triplicate determination ± SD. Different letters indicate the significant differences between stages according to Tukey’s test, *p* ˂ 0.05. DW: Dry Weight; MG3E: Malvidin-3-O-Glucoside Equivalent.

**Table 2:** Voltammetric behavior of the studied standard polyphenols in tartaric acid buffer (pH 3.6) with SWCNT-SPCE and GCE for a concentration of 0.1 mM.

Standards	Potential (mV)			
	SWCNT-SPCE (vs. Ag)		GCE (vs. Ag/AgCl-KCl 3M)	
	Ep,a1	Ep,a2	Ep,a1	Ep,a2
Catechin	132	468	483	826
Caffeic acid	139	/	445	/
Gallic acid	122	465	472	766
Oenin chloride	377	669	652	987
Rutin	201	460	260	898

GCE: Glassy Carbon Electrode; SWCNT-SPCE: Single Walled Carbon Nanotubes modified Screen Printed Carbon Electrodes.

**Table 3:** TPC and antioxidant capacities of skin and seed grape extracts of the three studied varieties at different stages of ripening by spectrophotometric methods.

	Skins			
	TPC (mg GAE/g DW)	DPPH (µmol TE/g DW)	ABTS (µmol TE/g DW)	FRAP (mmol Fe^+2^E/g DW)
**Merlot**				
**Green stage**	280 ± 39 ^a^	763 ± 67 ^a^	804 ± 37 ^a^	2781 ± 186 ^a^
**Close to veraison**	138 ± 12 ^b^	575 ± 46 ^b^	748 ± 41 ^a^	1925 ± 81 ^b^
**Veraison**	82 ± 11 ^b^	349 ± 24 ^c^	424 ± 11 ^b^	1036 ± 114 ^c^
**Maturity**	76 ± 9 ^b^	403 ± 28 ^c^	527 ± 80 ^b^	1180 ± 16 ^c^
**Tannat**				
**Green stage**	258 ± 21 ^a^	932 ± 120 ^a^	1211 ± 120 ^a^	2704 ± 431 ^a^
**Close to veraison**	188 ± 44 ^b^	647 ± 123 ^b^	1109 ± 188 ^a^	2322 ± 537 ^a^
**Veraison**	72 ± 14 ^c^	409 ± 54 ^b^	696 ± 79 ^b^	805 ± 8 ^b^
**Maturity**	111 ± 9 ^c^	528 ± 48 ^b^	612 ± 36 ^b^	1219 ± 39 ^b^
**Syrah**				
**Green stage**	212 ± 39 ^a^	853 ± 94 ^a^	843 ± 124 ^a^	2159 ± 432 ^a^
**Close to veraison**	103 ± 21 ^b^	674 ± 10 ^ab^	687 ± 141 ^ab^	1239 ± 251 ^b^
**Veraison**	85 ± 8 ^b^	639 ± 22 ^b^	529 ± 88 ^ab^	851 ± 29 ^b^
**Maturity**	63 ± 8 ^b^	557 ± 29 ^b^	357 ± 14 ^b^	780 ± 62 ^b^
	**Seeds**			
**Merlot**				
**Green stage**	867 ± 60 ^a^	3855 ± 413 ^a^	1681 ± 302 ^ab^	6047 ± 612 ^a^
**Close to veraison**	834 ± 7 ^a^	3998 ± 317 ^a^	1846 ± 123 ^a^	6006 ± 9928 ^a^
**Veraison**	805 ± 92 ^b^	3675 ± 172 ^a^	1663.92 ± 89 ^ab^	5436 ± 391 ^a^
**Maturity**	571 ± 23 ^b^	2876 ± 300 ^b^	1340.8 ± 67 ^b^	4683 ± 492 ^a^
**Tannat**				
**Green stage**	586 ± 57 ^ab^	3608 ± 201 ^ab^	1467 ± 266 ^ab^	4651 ± 726 ^ab^
**Close to veraison**	712 ± 69 ^a^	3875 ± 118 ^a^	1697 ± 45 ^a^	5557 ± 503 ^a^
**Veraison**	676 ± 18 ^a^	3706 ± 302 ^a^	1656 ± 137 ^a^	4201 ± 903 ^ab^
**Maturity**	489 ± 55 ^b^	3114 ± 127 ^b^	1240.95 ± 47 ^b^	3266 ± 300 ^b^
**Syrah**				
**Green stage**	556 ± 52 ^ab^	2677 ± 216 ^ab^	1171 ± 91 ^a^	3979 ± 2115 ^a^
**Close to veraison**	615 ± 21 ^a^	2915 ± 467 ^a^	1325 ± 46 ^a^	5386 ± 742 ^a^
**Veraison**	467 ± 6 ^b^	2366 ± 105 ^ab^	1079 ± 159 ^a^	4719 ± 639 ^a^
**Maturity**	454 ± 96 ^b^	1991 ± 211 ^b^	590 ± 186 ^b^	3460 ± 1065 ^a^

Values represent means of triplicate determination ± SD. Different letters indicate the significant differences between stages according to Tukey’s test, *p* ˂ 0.05. TPC: Total Phenolic Content; DPPH: 1,1-diphenyl-2-picrylhydrazyl free radical; ABTS: 2,2′-azino-bis(3-ethylbenzothiazoline-6-sulfonic acid)diammonium salt; FRAP: Ferric Reducing Antioxidant Potential; DW: Dry Weight; GAE: Gallic Acid Equivalent; TE: Trolox Equivalent; Fe^+2^E: Fe^+2^ Equivalent.

**Table 4:** Potential of peaks and cumulative peak areas for skins and seeds of Merlot, Tannat, and Syrah during ripening.

		Skins						
		Ep,a1 (mV)	Ep,a2 (mV)	Q_240mV_ (µC/g DW)	Q_520mV_ (µC/g DW)	Q_520mv_-Q_240mv_ (µC/g DW)	Q_800mV_ (µC/g DW)	Q_240mV_/Q_800mV_ (%)
**Merlot**	**Green stage**	137 ± 3 ^b^	391 ± 4 ^a^	44 ± 6 ^a^	153 ± 26 ^a^	110 ± 19 ^a^	262 ± 55 ^a^	17 ± 1 ^ab^
	**Close to veraison**	134 ± 2 ^b^	383 ± 4 ^a^	39 ± 3 ^a^	126 ± 14 ^a^	87 ± 11 ^a^	166 ± 1 ^b^	22 ± 3 ^a^
	**Veraison**	159 ± 5 ^a^	363 ± 2 ^b^	15 ± 1 ^b^	57 ± 2 ^b^	42 ± 1 ^b^	154 ± 4 ^b^	13 ± 1 ^b^
	**Maturity**	157 ± 3 ^a^	370 ± 1 ^b^	22 ± 1 ^b^	75 ± 5 ^b^	53 ± 4 ^b^	118 ± 7 ^b^	19 ± 1 ^a^
**Tannat**	**Green stage**	139 ± 5 ^b^	392 ± 5 ^a^	65 ± 8 ^a^	211 ± 20 ^a^	145 ± 13 ^a^	315 ± 36 ^a^	21 ± 1 ^a^
	**Close to veraison**	133 ± 1 ^b^	383 ± 2 ^b^	42 ± 10 ^b^	134 ± 37 ^b^	92 ± 27 ^b^	215 ± 5 ^b^	18 ± 6 ^a^
	**Veraison**	130 ± 9 ^b^	356 ± 3 ^c^	21 ± 6 ^c^	64 ± 18 ^c^	43 ± 12 ^c^	154 ± 24 ^b^	16 ± 8 ^a^
	**Maturity**	164 ± 2 ^a^	362 ± 1 ^c^	27 ± 5 ^bc^	105 ± 16 ^bc^	78 ± 11 ^bc^	174 ± 3 ^b^	18 ± 5 ^a^
**Syrah**	**Green stage**	137 ± 1 ^b^	383 ± 1 ^a^	36 ± 5 ^a^	119 ± 21 ^a^	83 ± 16 ^a^	172 ± 27 ^a^	21 ± 1 ^a^
	**Close to veraison**	126 ± 2 ^c^	377 ± 3 ^ab^	29 ± 5 ^ab^	91 ± 18 ^ab^	62 ± 13 ^ab^	152 ± 11 ^a^	18 ± 3 ^ab^
	**Veraison**	160 ± 5 ^a^	362 ± 3 ^bc^	21 ± 1 ^b^	77 ± 4 ^ab^	56 ± 3 ^ab^	141 ± 6 ^a^	16 ± 1 ^ab^
	**Maturity**	141 ± 3 ^b^	359 ± 2 ^c^	16 ± 3 ^b^	59 ± 5 ^b^	42 ± 3 ^b^	129 ± 1 ^a^	14 ± 2 ^b^
		**Seeds**						
**Merlot**	**Green stage**	129 ± 4 ^a^	390 ± 8 ^bc^	358 ± 36 ^a^	905 ± 90 ^a^	547 ± 54 ^a^	1232 ± 152 ^bc^	30 ± 3.64 ^a^
	**Close to veraison**	133 ± 3 ^a^	449 ± 4 ^a^	393 ± 24 ^a^	958 ± 53 ^a^	565 ± 29 ^a^	1407 ± 35 ^ab^	28 ± 1 ^ab^
	**Veraison**	132 ± 2 ^a^	397 ± 1 ^b^	379 ± 8 ^a^	944 ± 23 ^a^	564 ± 15 ^a^	1471 ± 65 ^a^	26 ± 0.63 ^ab^
	**Maturity**	128 ± 1 ^a^	380 ± 3 ^c^	252 ± 19 ^b^	639 ± 56 ^b^	387 ± 39 ^b^	1036 ± 94 ^c^	24 ± 0.47 ^b^
**Tannat**	**Green stage**	135 ± 3 ^a^	377 ± 6 ^b^	206 ± 29 ^b^	555 ± 64 ^b^	349 ± 35 ^b^	808 ± 112 ^b^	26 ± 1.63 ^a^
	**Close to veraison**	128 ± 1 ^a^	392 ± 6 ^ab^	319 ± 22 ^a^	827 ± 49 ^a^	508 ± 28 ^a^	1313 ± 52 ^a^	24 ± 1.08 ^a^
	**Veraison**	129 ± 3 ^a^	418 ± 20 ^a^	302 ± 16 ^a^	746 ± 49 ^a^	444 ± 33 ^a^	1112 ± 162 ^ab^	27 ± 2.45 ^a^
	**Maturity**	129 ± 4 ^a^	379 ± 2 ^b^	216 ± 7 ^b^	532 ± 20 ^b^	316 ± 16 ^b^	813 ± 61 ^b^	27 ± 1.14 ^a^
**Syrah**	**Green stage**	136 ± 3 ^a^	397 ± 5 ^c^	302 ± 18 ^b^	724 ± 36 ^b^	516 ± 179 ^ab^	1165 ± 26 ^ab^	26 ± 2.24 ^a^
	**Close to veraison**	127 ± 2 ^b^	438 ± 6 ^a^	388 ± 24 ^a^	937 ± 75 ^a^	549 ± 50 ^a^	1497 ± 15 ^a^	23 ± 0.33 ^a^
	**Veraison**	129 ± 1 ^b^	409 ± 4 ^b^	268 ± 20 ^b^	691 ± 80 ^b^	424 ± 30 ^ab^	1082 ± 44 ^ab^	25 ± 0.84 ^a^
	**Maturity**	126 ± 4 ^b^	391 ± 4 ^c^	177 ± 33 ^c^	463 ± 93 ^c^	286 ± 60 ^b^	818 ± 42 ^b^	24 ± 2.6 ^a^

Values represent means of triplicate determination ± SD. Different letters indicate the significant differences between stages according to Tukey’s test, *p* ˂ 0.05. DW: Dry weight.

**Table 5:** Pearson’s correlation coefficients of antioxidant capacity using spectrophotometric tests, electrochemical parameters, flavanols, and anthocyanins.

	**Skins**									
	**Folin**	**DPPH**	**ABTS**	**FRAP**	**Q**_**240mV**_	**Q**_**520mV**_	**Q**_**520mV**_ **-Q**_**240mV**_	**Q**_**800mV**_	**Flavanols**	**Anthocyanins**
**Folin**	1	0.83	0.80	0.94	0.88	0.90	0.90	0.84	0.93	−0.62
**DPPH**	0.83	1	0.75	0.79	0.81	0.82	0.81	0.69	0.86	−0.55
**ABTS**	0.80	0.75	1	0.84	0.89	0.86	0.85	0.69	0.86	−0.62
**FRAP**	0.94	0.79	0.84	1	0.86	0.87	0.87	0.75	0.94	−0.68
**Q**_**240mV**_	0.88	0.81	0.89	0.86	1	0.99	0.98	0.84	0.87	−0.60
**Q**_**520mV**_	0.90	0.82	0.86	0.87	0.99	1	1.00	0.85	0.86	−0.53
**Q**_**520mV**_ **-Q**_**240mV**_	0.90	0.81	0.85	0.87	0.98	1.00	1	0.85	0.85	−0.49
**Q**_**800mV**_	0.84	0.69	0.69	0.75	0.84	0.85	0.85	1	0.72	−0.50
**Flavanols**	0.93	0.86	0.86	0.94	0.87	0.86	0.85	0.72	1	−0.77
**Anthocyanins**	−0.62	−0.55	−0.62	−0.68	−0.60	−0.53	−0.49	−0.50	−0.77	1
	**Seeds**									
	**Folin**	**DPPH**	**ABTS**	**FRAP**	**Q**_**240mV**_	**Q**_**520mV**_	**Q**_**520mV**_ **-Q**_**240mV**_	**Q**_**800mV**_	**Flavanols**	
**Folin**	1	0.78	0.77	0.67	0.76	0.79	0.66	0.60	0.67	
**DPPH**	0.78	1	0.92	0.44	0.56	0.59	0.56	0.41	0.66	
**ABTS**	0.77	0.92	1	0.56	0.66	0.69	0.62	0.49	0.71	
**FRAP**	0.67	0.44	0.56	1	0.62	0.66	0.41	0.51	0.58	
**Q**_**240mV**_	0.76	0.56	0.66	0.62	1	0.99	0.89	0.88	0.80	
**Q**_**520mV**_	0.79	0.59	0.69	0.66	0.99	1	0.90	0.88	0.80	
**Q_520mV_-Q_240mV_**	0.66	0.56	0.62	0.41	0.89	0.90	1	0.79	0.74	
**Q**_**800mV**_	0.60	0.41	0.49	0.51	0.88	0.88	0.79	1	0.64	
**Flavanols**	0.67	0.66	0.71	0.58	0.80	0.80	0.74	0.64	1	

## Supplementary Materials

The following are available online at https://www.mdpi.com/2076-3921/9/9/800/s1. Table S1: Dates corresponding to the different stages of ripening for the three varieties Merlot, Tannat, and Syrah; Figure S1: The experimental electrochemical set up using GCE (A) and SWCNT (B) electrodes.
