# Supplementary material for: Voltammetric Behavior, Flavanol and Anthocyanin Contents, and Antioxidant Capacity of Grape Skins and Seeds during Ripening (Vitis vinifera var. Merlot, Tannat, and Syrah)
Source: Antioxidants (Basel). 2020 Aug 27;9(9):800. doi: 10.3390/antiox9090800 (PMC7554950; doi:10.3390/antiox9090800)
Supplement: Supplementary file 1 [file antioxidants-09-00800-s001.pdf]

Supplementary data

Table S1. Dates and BBCH-scale corresponding to the different stages of ripening for the 3 varieties Merlot, Tannat and Syrah

Stages of ripening	BBCH-scale	Merlot	Tannat	Syrah	Representative pictures
**Green stage**	77	28/06/2017	28/06/2017	28/06/2017	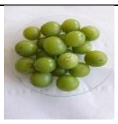
**Close to veraison**	79	11/07/2017	11/07/2017	11/07/2017	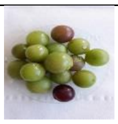
**Veraison**	81	25/07/2017	25/07/2017	25/07/2017	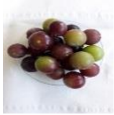
**Maturity**	89	14/09/2017	14/09/2017	14/09/2017	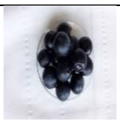

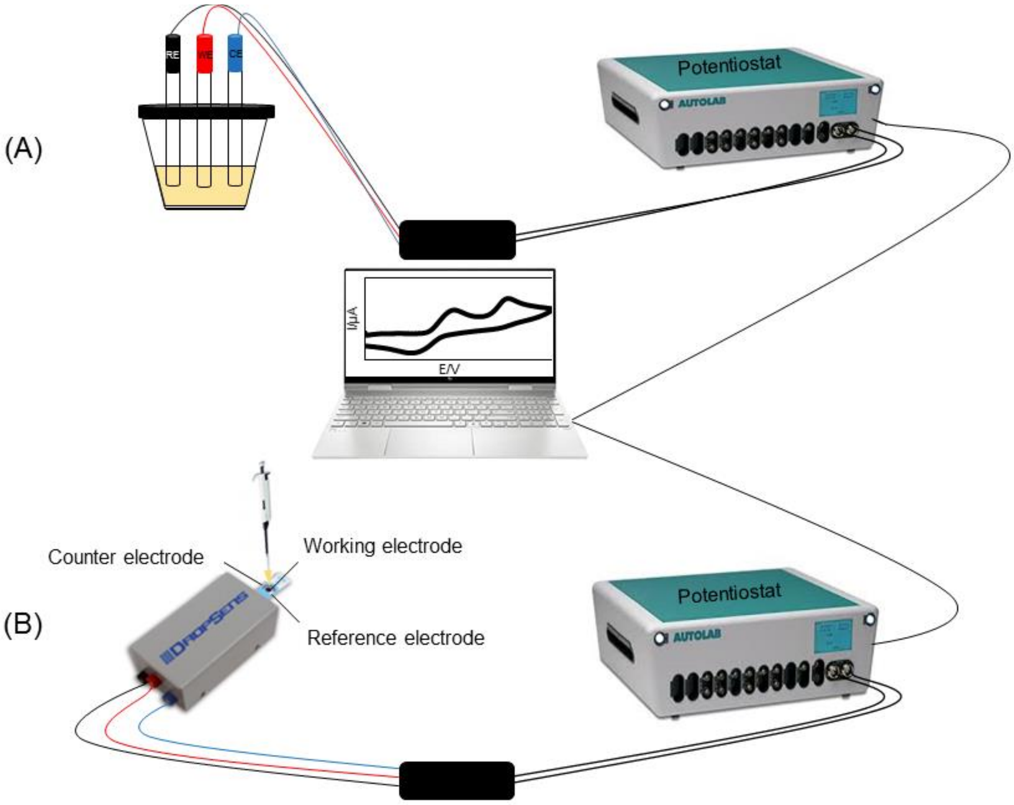

Figure S1. The experimental electrochemical set up using GCE (A) and SWCNT (B) electrodes.
